# A Study on the Condensation Reaction of 4-Amino-3,5-dimethyl-1,2,4-triazole with Benzaldehydes: Structure and Spectroscopic Properties of Some New Stable Hemiaminals

**DOI:** 10.3390/molecules200917109

**Published:** 2015-09-17

**Authors:** Katarzyna Wajda-Hermanowicz, Damian Pieniążczak, Aleksandra Zatajska, Robert Wróbel, Krzysztof Drabent, Zbigniew Ciunik

**Affiliations:** Faculty of Chemistry, University of Wrocław, F. Joliot-Curie 14, Wrocław 50-383, Poland; E-Mails: alecempire@o2.pl (D.P.); aleksandra.zatajska@chem.uni.wroc.pl (A.Z.); robert.wrobel@chem.uni.wroc.pl (R.W.); krzysztof.drabent@chem.uni.wroc.pl (K.D.); ciunik@wchuwr.pl (Z.C.)

**Keywords:** 3,5-dimethyl-1,2,4-triazole 4-amine, stable hemiaminals, chemical reactivity, Schiff bases, X-ray structures

## Abstract

Studies on the stable hemiaminals and Schiff bases formation in the reaction of substituted benzaldehydes with primary 3,5-dimethyl-1,2,4-triazole 4-amine were carried out under neutral conditions. These products were investigated by IR, Raman, MS, ^1^H- and ^13^C-NMR spectra as well as by X-ray crystallography. The effect of reaction conditions: temperature, polarity of the solvents utilized, substrate concentration and the *ortho* and *para* benzaldehyde substituents on the yield of products was also examined.

## 1. Introduction

1,2,4-Triazoles and their derivatives have attracted significant attention in several different areas. These nitrogen-rich compounds represent one of the most biologically active classes of the chemical species. This arises from their ability to bind to a variety of enzymes and receptors in biological system via diverse non-covalent interactions [1,2,3]. The antimicrobial activity 3,5-dimethyl-1,2,4-triazole derivatives has been reported [4]. The Schiff bases obtained from 4-amino-3,5-dimethyl-1,2,4-triazole inhibit endocytosis [5] and in copper(II) complexes inhibit protein tyrosine phosphatases [6]. Additionally, 4-Amino-3,5-dimethyl-1,2,4-triazole is a very interesting bridging ligand. It coordinates with Mn(II), Co(II) [7], Cu(II) [8] and mixed valence cobalt [9] forming trinuclear coordination compounds which exhibit very interesting magnetic properties. Polymeric Ag(I) complexes with amino-triazole acting as a tridentate-N donor ligand were also obtained [10]. Trinuclear azide complexes of Cu, Co, Ni, Zn, Mn and Cd were investigated as energetic materials [11]. The inhibitive effect of 3-methyl-4-amino-1,2,4-triazole on the corrosion of copper-nickel alloys has been reported [12]. Furthemore, 4-amino-1,2,4-triazoles and its 3,5-dimethyl derivative readily react with alkylated agents at the N-1 position forming low melting points ionic liquid salts [13].

It is well known that the primary and secondary amines react by nucleophilic addition with carbonyl compounds to give intermediate tetrahedral addition products called hemiaminals [14] as a first step of condensation reaction [15]. The next step is the dehydration of that compound which leads to the formation of stable imines, enamines, hydrazones and related compounds [16]. Typically, the hemiaminals are short–lived species. They are sometimes detected by spectroscopic methods: IR [17], NMR [18,19,20] and by X-ray observation [21]. The tetrahedral carbinolamine group can be stabilized also by metal ions. Nitrogen-containing aromatic heterocyclic aldehydes react with di-(2-picolyl)amine in presence of Zn^2+^ salts to form labile tris-(2-picolyl) hemiaminal zinc complexes [22]. Rhodium (III) complexes of *o*-diphenylphosphinobenzaldehyde react with dihydrazones to give ionic species with a new tridentate PNN–hemiaminal type ligand [23].

One of the stable hemiaminals was obtained as a solid in the reaction between trifluoroacetaldehyde and a secondary amine, *N*-benzyl piperazine. Fluoral acts as an efficient nucleophilic trifluoromethylating agent towards non-enolizable carbonyl compounds under mild conditions [24]. The stable-in-solution hemiaminals were prepared in the reactions of methyl 3,3,3-trifluoropyruvate and benzylic monoamines and diamines. A similar product obtained from aniline was stable only under inert atmosphere but not in solutions [25]. Hexafluoroacetone reaction with 2-(aminomethyl)aniline also results in a stable tetrahedral product with benzylic amino group [26]. Another stable hemiaminal was obtained from 4-cyclohexyl-3-thiosemicarbazide and di-2-pyridyl ketone [27]. In this case, the formation of a carbinolamine was strongly dependent on the intramolecular hydrogen interactions between N–H∙∙∙N and O–H∙∙∙N (py) atoms. The addition of LiBH_4_ or the Grignard nucleophilic reagents MeMgCl and PhMgBr to amides is another way of making stable hemiaminals. Aromatic, alkyl and α,β-unsaturated *N*-acylpyrrole derivatives or one-carbon bridged amides allow one to obtain stable products with good yields [28,29]. Recently, the stable hemiaminals coming from the reaction between 4-amino-1,2,4-triazole and nitro-substituted [30] or cyano-substituted [31] benzaldehydes in acetonitrile under neutral conditions were also obtained. Eleven of them were structurally and spectroscopically characterized.

The present paper describes a novel application of 4-amino-3,5-dimethyl-1,2,4-triazole in the preparation of stable hemiaminals. The effects of temperature, solvent, structure and concentration of the reagents on the product yields and stability are considered. Our results can contribute to a better understanding of the mechanism of hemiaminal formation from aminotriazoles and benzaldehydes.

## 2. Results and Discussion

**Scheme 1:**
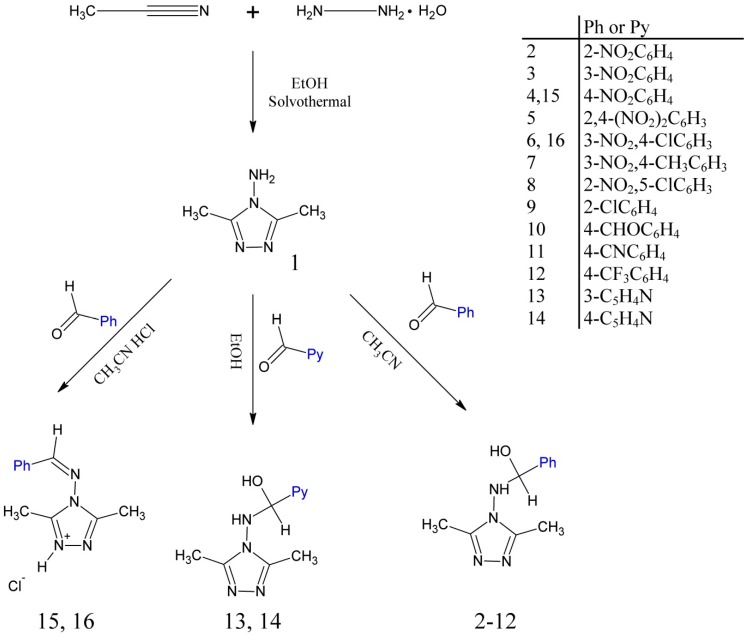
Synthetic pathway for preparation of compounds **1** to **16**.

By using a published method, 4-amino-4*H*-3,5-dimethyl-1,2,4-triazole (**1**) was obtained from one-pot solvothermal reaction of acetonitrile with hydrazine hydrate [32]. The syntheses of (aryl)(3,5-dimethyl-4*H*-1,2,4-triazole-4-ylamino)methanol (**2**–**14**) and *N***-**benzylidene-4*H*-3,5-dimethyl-1,2,4-triazole-4-amine (**15**, **16**) derivatives were accomplished according to the reaction outlined in Scheme 1. It should be noticed that the stable hemiaminals are formed only from aromatic aldehydes containing electron-withdrawing groups or atoms.

### 2.1. X-ray Diffraction

**Figure 1:**
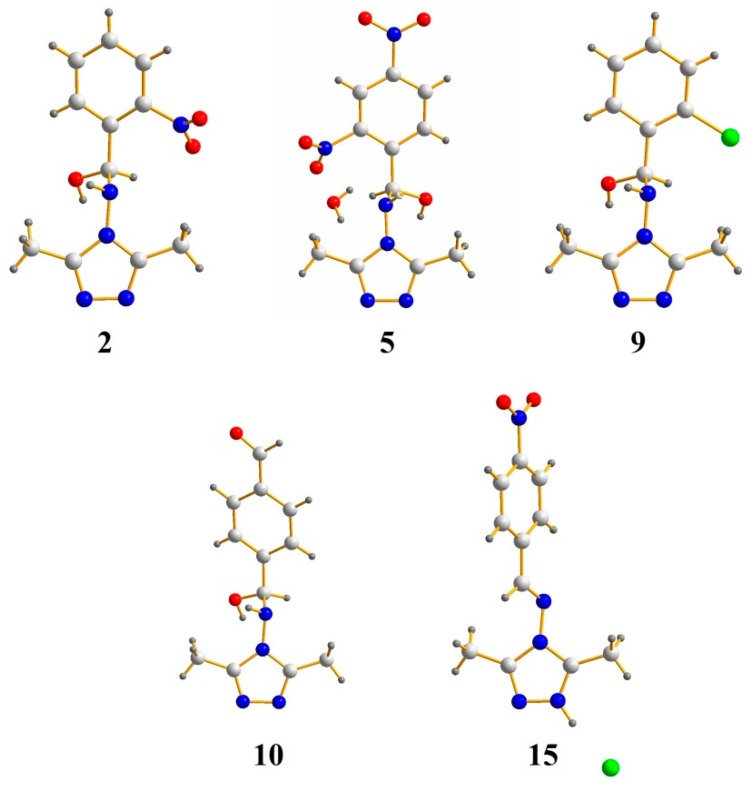
Molecular structure of compounds **2**, **5**, **9**, **10** and **15**.

Suitable crystals for X-ray diffraction were obtained for compounds **2**, **5**, **9**, **10** and **15** (Figure 1). The molecular structure consists of two phenyl and triazole aromatic rings connected with the C_1Ph_-C-N_4Tr_-N_3Tr_ sequence. In the hemiaminals, the C and N_4Tr_ atoms are tetrahedral with sp^3^ hybridization, which enables formation of four stereoisomers (RS, SR, RR and SS). For imine **15**, there is a C=N_4Tr_ double bond with sp^2^ hybridization. The general atom numbering and selected parameters are summarized in Table 1.

**Table 1:** Selected geometrical parameters for hemiaminals (**2**, **5**, **9**, **10**) and imine (**15**). 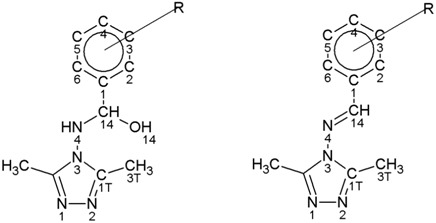

R	Bond Lengths (Å)						Torsion Angle (°)	Dihedral Angle (°)
	C_1_-C_14_	C_14_-N_4_	N_4_-N_3_	C_14_-O_14_	C_1t_-C_3t_	N_1_-N_2_	C_1_-C_14_-N_4_-N_3_	Phenyl-Triazole
2-NO_2_ (**2**)	1.529(2)	1.514(3)	1.425(2)	1.363(3)	1.435(2)	1.360(3)	177.1(1)	19.99(1)
2,4-(NO_2_)_2_ (**5**)	1.526(3)	1.461(2)	1.406(2)	1.397(3)	1.481(3)	1.396(4)	−176.3(2)	21.27(1)
2-Cl (**9**)	1.513(4)	1.467(4)	1.410(4)	1.425(4)	1.484(4)	1.403(3)	179.0(2)	9.9(2)
4-CHO (**10**)	1.510(2)	1.482(2)	1.409(2)	1.401(2)	1.478(2)	1.399(2)	174.3(1)	28.8(2)
4-NO_2_ (**15**)	1.465(2)	1.265(2)	1.418(2)	-	1.468(3)	1.367(2)	179.1(1)	70.48(2)

**Figure 2:**
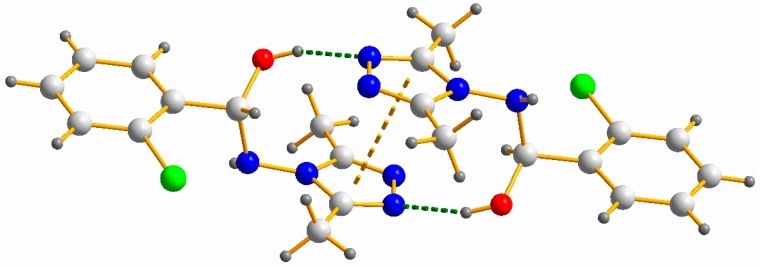
View of a part of the crystal structure of **9** showing the formation of a hydrogen-bonded dimer and the π-π interaction of triazole rings.

The hemiaminal molecules in **2**, **5**, **9** and **10** form a centrosymmetric (RS-SR) dimer linked by a O–H∙∙∙N_1Tr_ hydrogen bond (see Figure 2). A strong π-π interaction involving pairs of triazole rings additionally stabilizes the dimers (Table 2).

**Table 2:** Hydrogen bond and weak interactions [Å, °] in hemiaminals (**2**, **5**, **9**, **10**).

R	O_14_–H∙∙∙N_2_				C_g1_-C_g2_^a^
	D–H	H∙∙∙A	D∙∙∙A	<D–H∙∙∙A	
2-NO_2_ (**2**)	0.90(2)	1.87(2)	2.750(2)	169(3)	3.488(1)
2,4-(NO_2_)_2_ (**5**)	0.85(4)	2.00(4)	2.811(3)	161(3)	3.371(1)
2-Cl (**9**)	0.86(4)	1.96(4)	2.771(4)	156(4)	3.501(2)
4-CHO (**10**)	0.94(1)	1.77(3)	2.760(1)	175(2)	3.272(2)

^a^: C_g_ for centroid of triazole ring.

The structures presented above differ from those obtained by us earlier [30,31] where hemiaminals derived from 3,5-unsubstituted triazoles occur in two conformers: stretched (with configuration RS or SR) and twisted (RR or SS). Furthermore, in the title compounds the centrosymmetric dimers are observed whereas hemiaminals described by us previously [30,31] form infinite polymeric chains or noncentrosymmetric dimers.

### 2.2. Spectral Studies

The characteristic IR and Raman spectral bands of hemiaminals are given in Table 3. Characteristic strong ν(C=O) stretching vibration at about 1700 cm^−1^ observed in the infrared spectra in aromatic aldehydes, as well as bands observed at 3243 cm^−1^, 3152 cm^−1^ and 1650 cm^−1^ which were assigned to the ν_as_NH_2_, ν_s_NH_2_ and σ,ωNH_2_ vibrations [33] respectively for 4-amino-3,5-dimethyl-1,2,4-triazole, disappear after condensation reaction. A comparison between the NH and OH stretching bands, which were observed for hemiaminals, shows that they appear in the same spectral region. In the IR spectra, the strong OH bands sometimes mask the weaker NH absorption, but in the Raman spectra, the OH bands are very weak [34]. The –OH stretching vibration of the hydroxyl group is observed as a distinct peak at about 3200–3300 cm^−1^ in the IR spectrum. Their values increase with decreasing d_C-O_ bond distance (Table 1). The intramolecular hydrogen bonding interactions of C–OH with N_2tr_ observed in the crystal structures are confirmed by an additional broad shallow –OH stretching peak observed at about 3100 cm^−1^. The band appearing in the Raman spectra at about 3100 cm^−1^ is assigned to the stretching vibration of –NH.

**Table 3:** Selected spectral data of hemiaminals R_1_C*H(OH)NHR_2_.

R_1_	Vibration Frequencies (cm^−1^)			^1^H-NMR δ (ppm), *J* (Hz)					^13^C-NMR (ppm)
	ν_OH_^a^	ν_OH.N_^a^	ν_NH_^b^	δ_(NH)_	δ_(OH)_	δ_(CH)_	J_CH-NH_	J_CH-OH_	δ(C^*^)
2-NO_2_ C_6_H_4_ (**2**)	3308	3114	3090	7.15	6.91	5.96	8.12	5.33	79.2
3-NO_2_ C_6_H_4_ (**3**)	3312	3115	3090	7.19	6.79	5.62	7.17	5.78	83.2
4-NO_2_ C_6_H_4_ (**4**)	3304	3079	3100	7.17	6.76	5.58	7.18	5.67	83.4
2,4-(NO_2_)_2_ C_6_H_3_ (**5**)	3305	3106	3090	7.33	7.26	6.00	8.35	5.01	79.1
3-NO_2_,4-Cl C_6_H_3_ (**6**)	3261	3105	3080	7.20	6.86	5.57	7.44	5.72	82.7
3-NO_2_,4-CH_3_ C_6_H_3_ (**7**)	3309	3105	3070	7.12	6.70	5.54	6.87	5.69	82.6
2-Cl, 5-NO_2_ C_6_H_3_ (**8**)	3309	3070	3081	7.26	7.02	5.77	6.99	5.48	80.0
2-Cl C_6_H_4_ (**9**)	3265	3124	3081	7.05	6.60	5.76	6.29	5.15	80.8
4-CHO C_6_H_4_ (**10**)	3291	3077	3080	7.12	6.64	5.55	6.87	5.53	83.9
4-CN C_6_H_4_ (**11**)	3280	3082	3084	7.13	6.70	5.53	7.06	5.72	83.6
4-CF_3_ C_6_H_4_ (**12**)	3284	3126	3082	7.11	6.66	5.55	6.87	5.53	83.7
3-C_5_H_4_N (**13**)	3202	3125	3078	7.13	6.25	5.47	6.87	5.91	82.2
4-C_5_H_4_N (**14**)	3191	3122	3107	7.15	6.70	5.47	7.44	5.72	83.2

^a^: IR; ^b^: Raman.

The NMR spectra were obtained in the DMSO solution. DMSO is one of the most polar and aprotic solvents with a high dielectric constant and, due to this, properties of the dissolving species do not come together to agglomerate. For that reason, the hydrogen bonds observed in the solid state are not detected in the ^1^H-NMR spectra. In the ^1^H-NMR spectra of the compounds **2**–**16**, the singlet at δ 5.73 ppm, assigned to the NH_2_ protons of the starting compound **1**, disappeared and additional resonances assigned to the C–NH–N, C–OH and CH–N for **2**–**14** (Table 3) and –CH=N– (δ = 9.17 and 9.16) for **15** and **16** were detected which confirmed the condensation between the amino and the carbonyl groups.

The proton signals of the methyl triazole substituents (**1**–**16**) were observed as singlet at δ 2.25 ppm for amine and were shifted to a lower field in the order: amine-hemiaminal-schiff base (δ(CH_3_) = 2.25; 2.34 and 2.59 ppm for **1**, **4** and **15**, respectively). The ^13^C-NMR spectra of R_1_C*H(OH)NHR_2_ showed a characteristic signal δC* at 79.1–80.8 ppm for *ortho*, at 82.2–83.3 ppm for *meta* and at 83.2–83.9 ppm for *para* substituted R_1_ aromatic ring.

### 2.3. Hemiaminal Stability in Solution

**Scheme 2:**
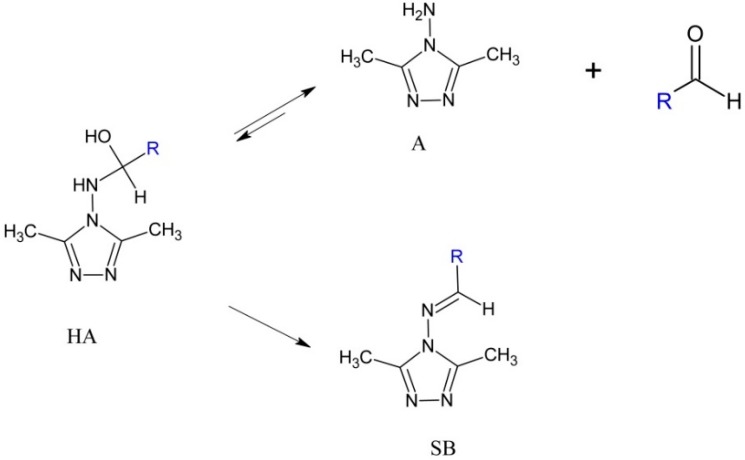
Hemiaminal decomposition reactions.

The hemiaminal under investigation were stable for a long time in the crystalline form. This observation does not apply to the compounds in solution. The time dependent changes in the ^1^H-NMR spectra were used to determine the decomposition of the hemiaminals in DMSO solution at room temperature (Scheme 2).

**Figure 3:**
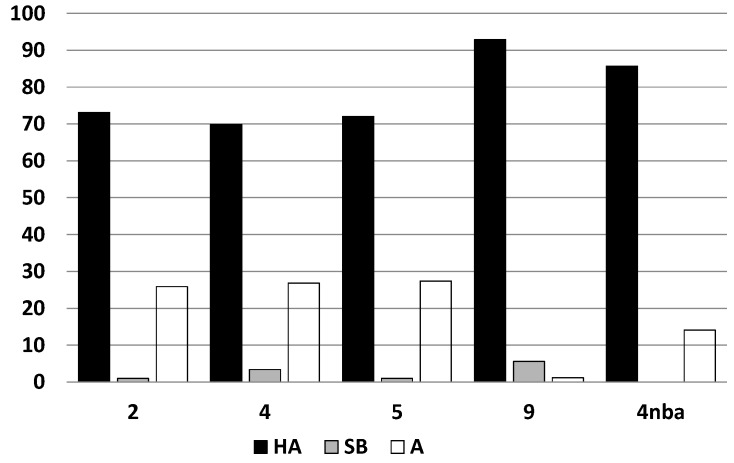
Conversion (%) of the hemiaminals (**HA**) **2**, **4**, **5**, **9** and **4nba** to substrates (**A**) and Schiff bases (**SB**) after 120 h in DMSO solution.

**Figure 4:**
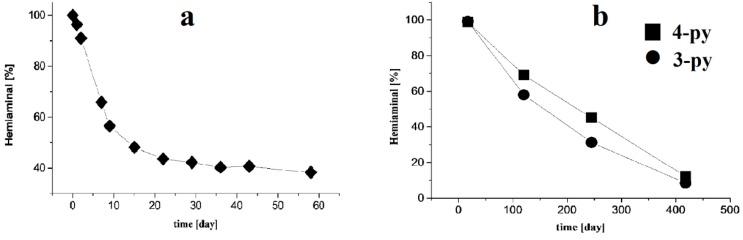
Decomposition of the hemiaminals (**a**) **4nba**; (**b**) **13** and **14** in DMSO solution as the function of time.

Compounds **2**, **4**, **5** and **9** decompose slowly mostly to substrates (Figure 3), similar to the hemiaminal obtained from the 4-nitrobenzaldehyde and 4-amino-1,2,4-triazole (**4nba**, see Figure 4a).

A greater stability in solution is observed for compounds obtained from the 3- and 4-pyridinecarboxaldehyde. Even after one year, **13** and **14** are detected in solution in the amount of about 20% (Figure 4b). In contrast to the 3- and 4-pyridinecarboxaldehyde, the compound obtained by the condensation of 2-pyridine derivative in EtOH give solely the Schiff base **17a**. The products obtained from the condensation performed in hexane solution were a mixture of the hemiaminal **17** and Schiff base **17a** in the molar ratio 1:2. In DMSO solution, hemiaminal **17** quickly converted to the Schiff base **17a**.

These observations agree well with the theoretically examined Schiff base formation mechanism from benzaldehyde and 4-amine-4*H*-1,2,4-triazole [35]. The reaction takes place in two steps. In the first step, the hemiaminal is formed. The formation of Schiff base through the water molecule elimination requires an internal equilibrium between the twisted conformation of hemiaminals. The ^1^H-NMR spectral data for all stable hemiaminals obtained from 4-amino-3,5-dimethyl-1,2,4-triazole showed that they are stretched conformers. The coupling of NH protons with vicinal CH protons is about 7 to 8 Hz (Table 3). The coupling constant ^3^*J*_(CH-NH)_ for 2-pyridinyl hemiaminal **17** is smaller (4.96 Hz) which indicates that the twisted isomer dominates in solution [36].

### 2.4. Hemiaminal Formation—Effect of Substituents.

To gain a better understanding of the substrate structure effect on the hemiaminal formation, a series of 2- and 4-substituted benzaldehydes was examined, focusing on their reaction with **1** in a 1:1 stoichiometry in CH_3_CN solution. The reaction mixtures were stirred at 50 °C over 9 h. After solvent evaporating, the remaining solids were investigated by ^1^H-NMR in DMSO solution.

**Figure 5:**
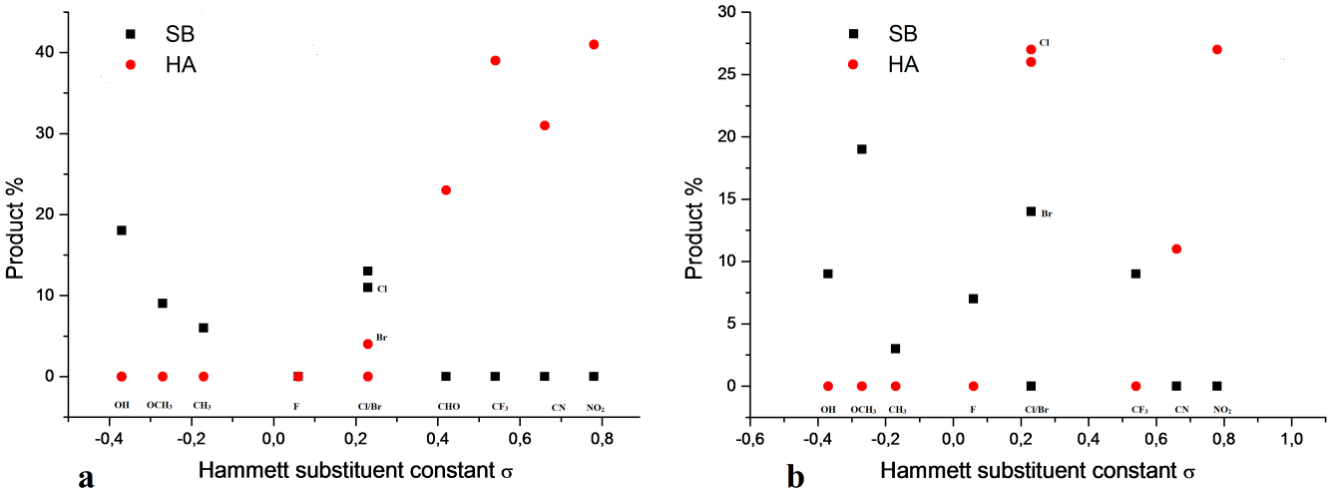
Variation of the hemiaminal (HA ●) and Schiff base (SB ■) formation from 4-amino-3,5-dimethyl-1,2,4-triazole and (**a**) *para* and (**b**) *ortho* substituted benzaldehydes as a function of the corresponding substituent constant [38]. All reaction were performed using 0.172 mmol of substrates in CH_3_CN (2 mL) at 50 °C. Product yields were obtained from the CH_3_^1^H-NMR signals in the region of 2.00–2.50 ppm.

The good correlation between the imine and hemiaminal formation and electronic effects of the substituents is observed only for *para* derivatives (Figure 5a). From the theoretical studies [35,37] it is known that the N–H amine bond is broken first and then the hydrogen atom is transferred to the aldehyde O atom forming an O–H bond. Subsequently, the C–N bond is formed. It seems that the hemiaminal formation must be dependent on the carbonyl C atom electrophilicity. Benzaldehydes containing electron-withdrawing (-R) substituents reduce the hemiaminal formation in order: NO_2_ > CN > CF_3_ > CHO > H. Opposite to this, in the case of the substituents containing electron-donating groups (+R), the formation yield increases in order: OH < OCH_3_ < CH_3_ < F < Cl < Br < H. The next step of reaction is water molecule elimination from hemiaminal.

The C–OH bond is broken first. Then, the N–H bond is broken and finally an imine and water are formed. It seems that the stability of the C–OH bond is also dependent on the phenyl ring substituent and this relation is opposite to that described above for hemiaminal formation. The C–O bond is being broken more easily for +R than for −R substituents.

The effect of *ortho* substituents on the condensation product reaction is more complex (Figure 5b) than for *para* substituents and could not be explained by the differences in electrophilicity of the carbonyl C atom.

### 2.5. Hemiaminal Formation—Solvent Effect

The condensation reaction of 2-nitrobenzaldehyde with 4-amino-3,5-dimethyl-1,2,4-triazole was studied in 12 different organic solvents. The solvent effect on the reaction rate and efficiency was investigated by the ^1^H-NMR spectroscopy (Table 4). The results indicate a higher hemiaminal content in apolar aprotic solvents than in dipolar aprotic media. The hemiaminal yield increases with solvent hydrophobicity, whereas a polar solvent shifts the equilibrium towards the Schiff base formation. Although, at first sight, it is surprising that increasing solvent polarity diminishes the hemiaminal content, this is understandable in terms of changing substrates and products dipole moment. The rate of the first step of condensation decreases with increasing solvent polarity because the activated complex must be less dipolar than the reactants. It means that the dipole moment of the activated complex should be less than the sum of the reactant dipole moment [39]. From the theoretical study [35], it is known that the hemiaminal, as an intermediate of the condensation, is non-ionic. On the other side, due to the strong intermolecular hydrogen interaction, the existence of dimers is possible, which can reduce the polarity of a hemiaminal. The strong influence of the solvent on the second step of the formation of Schiff base and elimination of the water molecule was also observed. The rate is slowest in polar aprotic solvents with high dipole moment. It seems that the activated complex, which leads to the Schiff base, appears to be less dipolar and hence less strongly solvated. In the aprotic electron-pair donor solvents with small dipole moments, the rate of this step is faster. In the hydrogen bonding solvent such as water or *iso*-propanol, the hemiaminal formation can proceed via a zwitterionic intermediate. The calculations of zwitterion formation between methylamine and formaldehyde have been performed [40] and found that two water molecules reduce the reaction barriers of proton-transfer step [41]. As indicated in Table 4, the water role in the 2-nitro hemiaminal formation in acetonitrile solution is not restricted only to solvent effects [42], as water also acts as a reactive species. The catalytic properties of water molecules in this reaction were thought to be essential in order to facilitate the nucleophilic attack of the amine on the carbonyl group and the proton transfers from amine to water molecule and from water to aldehyde oxygen. The rate for the first step of condensation reaction of **1** with 2-nitrobenzaldehyde depends on the water content in acetonitrile and maximum rate acceleration was observed at 15% by volume water in acetonitrile (Table 4).

### 2.6. Hemiaminal Formation—Benzaldehyde Concentration and Temperature Effect

**Figure 6:**
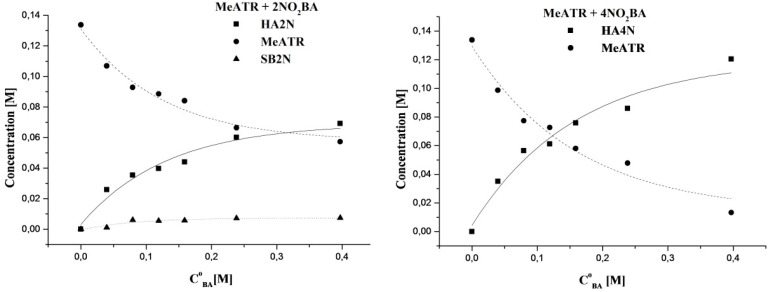
Variation of the 4-amino-3,5-dimethyl-1,2,4-triazole (MeATR ●), hemiaminal (HA ■) and Schiff base (SB ▲) concentration as a function of the initial 2 and 4-nitrobenzaldehyde concentration in the reaction of MeATR with benzaldehydes in CH_3_CN solution at 50 °C after 9 h. Product concentrations were obtained from the CH_3_^1^H-NMR signals in the region of 2.00–2.50 ppm.

The 4-amino-3,5-dimethyl-1,2,4,-triazole is in dynamic equilibrium with the reactant aldehydes. In order to determine the experimental conditions that favor the shift of the equilibrium toward the hemiaminal as a product, the effects of temperature and benzaldehyde concentration were determined using 2- and 4-nitro substituted benzaldehydes. As can be seen (Figure 6), the highest hemiaminal yield was obtained in the upper range of the aldehyde to amine molar ratio.

**Table 4:** Solvent effect on the hemiaminal (**HA**) and Schiff base (**SB**) formation from 2-nitrobenzaldehyde and 4-amino-3,5-dimethyl-1,2,4-triazole.

Solvent	HA ^a^	SB ^a^	K ^b^
*n*-Hexane	70	4	17.5
Cyclohexane	47	11	4.3
CHCl_3_	47	14	3.4
Toluene	33	8	4.1
CH_2_Cl_2_	28	16	1.8
CH_3_CN	27	4	6.8
DMSO	48	12	4.0
Pyridine	35	13	2.7
Triethylamine	25	19	1.3
THF	30	26	1.2
1,4-Dioxane	6	44	0.1
2-Propanol	38	12	3.2
H_2_O	33	8	4.1
**H_2_O/CH_3_CN (V:V)**			
1.5:0.5	41	10	4.1
1.0:1.0	42	7	6.0
0.5:1.5	41	6	6.8
0.4:1.6	44	6	7.3
0.3:1.7	51	5	10.2
0.2:1.8	51	6	8.5
0.1:1.9	54	7	7.7

^a^: (I_X_/I_A_ + I_HA_ + I_SB_) × 100 where I is integrated peak intensities of the CH_3_ signals in ^1^H-NMR spectrum (I_A_-amine, I_HA_-hemiaminal and I_SB_-Schiff base); ^b^: K = HA/SB.

In Table 5, the values of molar ratio K calculated for the formation of hemiaminal **2** and respective Schiff base (from the amine **1** and 2-nitrobenzaldehyde) in acetonitrile at different temperatures are presented. The results show that the temperature increase favors the imine formation. However, it must be noticed that the summary yield of products (HA + SB) at all temperatures is about 30%. This probably indicates that the first step of the reaction—the hemiaminal formation—is a reversible and exothermic process [43]. The second step of Schiff base formation is endothermic.

**Table 5:** The molar ratio of hemiaminal to imine (K) values calculated at different temperatures for the reaction of **1** with 2-nitrobenzaldehyde in CH_3_CN.

Temperature °C	40	50	60	70	80
K = I_HA_/I_SB_^a^	8.3	6.8	5.3	3.9	2.4

^a^: I is integrated peak intensities of the CH_3_ signals in ^1^H-NMR spectrum.

### 2.7. Hemiaminal-Aldehyde Interchange Reaction

**Scheme 3:**
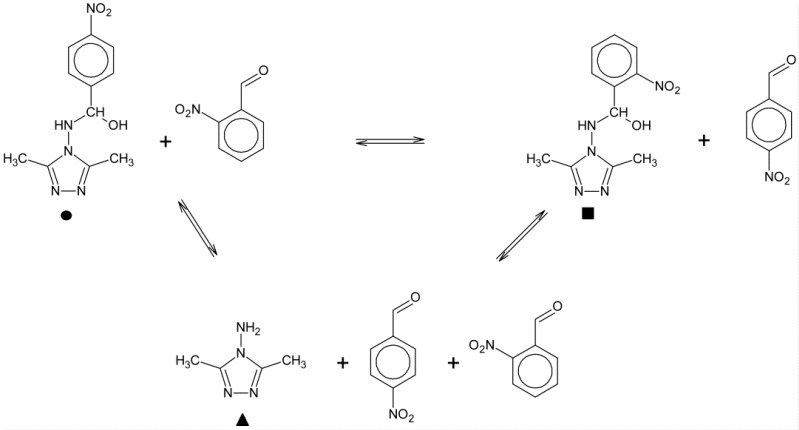
Hemiaminal-aldehyde interchange reaction.

**Figure 7:**
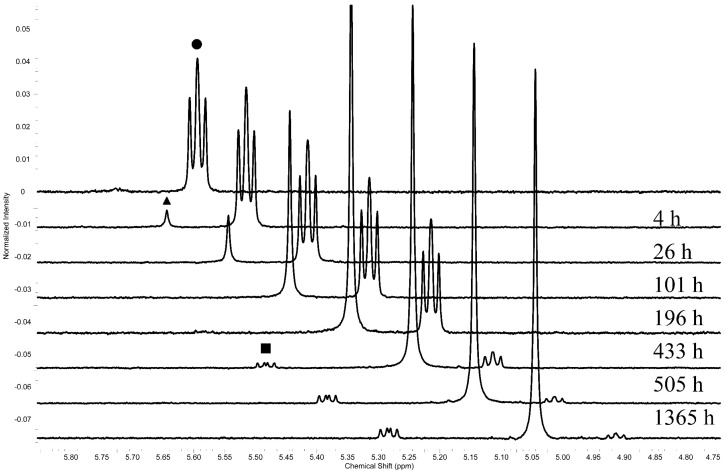
^1^H-NMR monitoring of the C*H hemiaminal (●—4-NO_2_, ■—2-NO_2_) and NH_2_ (▲—amine **1**) protons) during the metathesis reaction of 4-nitrobenzaldehyde and 2-nitrobenzaldehyde (Scheme 2) (DMSO-*d*_6_, 25 °C).

Finally, the aromatic aldehyde interchange reaction in DMSO solution at room temperature was studied by the ^1^H-NMR. The spectra in Figure 7 show that upon addition of 2-nitrobenzaldehyde and 4-nitro substituted hemiaminal **4** (12.5 mM) in molar ratio 2:1, respectively, in DMSO-*d*_6_ at 25 °C, a new signal appears in the hemiaminal proton region. The above experiments also show that the metathesis reaction is occurring quite slowly and that the first step of this process is the hemiaminal disintegration to amine and aldehyde (Scheme 3).

## 3. Experimental Section

### 3.1. Materials and Physical Measurements

The reagents and solvents employed were commercially available and used as received without further purification. Elemental analyses were carried out with a CHNS Vario EL III analyzer (Elementar Analysensystem GmbH, Hanau, Germany). The NMR spectra were recorded on a Bruker 300 or 500 MHz spectrometer (Bruker, Poznań, Poland) using solvent as an internal standard. The mass spectra of electrospray ionization (ESI)-MS were obtained on MicrOTOF-Q mass spectrometer (Bruker). The Fourier transform IR spectra were recorded from KBr pellets in the range of 400–4000 cm^−1^ on a Bruker IFS 66 FT-IR (Bruker). The Fourier-Transform Raman Nicolet Magna 860 FTIR/FT Raman spectrometer (Spectro-Lab, Warszawa, Poland) was used for the Raman spectral measurements at room temperature.

4-amino-3,5-dimethyl-1,2,4-triazole (MeATR) was synthesized in accordance with the published procedure and checked with ^1^H-NMR spectra and elemental analysis [32].

### 3.2. X-ray Crystallography

Single crystal X-Ray diffraction data were collected at Xcalibur four-circle diffractometer (Wrocław, Poland) with graphite monochromated Mo Kα radiation (λ = 0.71073 Å) at 298 K (**2**, **5**, **15**) and 100 K (**9**, **10**) using an Oxford Cryosystem adapter [44] and CC. Data collection and data reduction CrysAlisPro, Agilent Technologies [45] program used. The structures were solved by direct methods with SHELXS and was refined by a full-matrix least squares method using SHELXL97 programs [46]. CCDC 1412796-1412800 contains the supplementary crystallographic data for this paper. These data can be obtained free of charge via http://www.ccdc.cam.ac.uk/conts/retrieving.html (or from the CCDC, 12 Union Road, Cambridge CB2 1EZ, UK; Fax: +44-1223-336033; E-Mail: deposit@ccdc.cam.ac.uk).

### 3.3. Synthesis of Hemiaminals **2**–**14**

Compounds **2**–**12** were synthesized according to the following general procedure. A mixture of equimolar amounts (0.54 mmol) of MeATR (**1**) and a suitable aldehyde ArCHO (in molar ratio 1:1) were dissolved in acetonitrile (5 mL) and refluxed for 3 h. After removing volatile components, the raw solid products was washed with cold acetonitrile and dried in air. Crystals of four hemiaminals were obtained upon slow evaporation of the solvent from the reaction mixtures.

*(4H-3,5-Dimethyl-1,2,4-triazole-4-ylamino)(2-nitrophenyl)methanol* (**2**): Yield 96%. Anal. Calc. (%) for C_11_H_13_N_5_O_3_: C, 50.18; H, 4.98; N, 26.61. Found: C, 49.85; H, 4.28; N, 25.61. IR (KBr, cm^−1^): 373 w, 393 w, 413 w, 501 m, 512 w, 572 m, 587 vs, 607 m, 624 m, 663 s; 685 m; 719 vs; 751 s; 785 vs; 858 s; 874 m; 892 m; 959 w; 978 w; 1025 m; 1040 s; 1061 vs, 1101 vs, 1144 w, 1164 m, 1193 s, 1248 m, 1314 m, 1361 vs, 1417 vs, 1445 s, 1474 m, 1498 s, 1531 vs, 1566 s, 1612 m, 2882 s, 3114 s, 3308 vs. Raman (cm^−1^): 210 vw, 275 vw, 307 vw, 336 vw, 398w, 415w, 590 w, 622 vw, 625w, 666 vw, 705 w, 765 vw, 857 s, 892 vw, 1040 s, 1060 vw, 1100 vw, 1150 w, 1170 w, 1190 w, 1360 vs, 1450 vw, 1530 w, 1580 w, 1610 w, 2940 vw, 2980 vw, 3000 vw, 3040 vw, 3080 w, 3090 m, 3310 vw. MS (ESI, *m*/*z*): 264.1 [M + H]^+^; 286.1 [M + Na]^+^; 302.1 [M + K]^+^, 549.2 [2M + Na]^+^. ^1^H-NMR (DMSO-*d*_6_, 298 K, ppm, 500 MHz): δ = 7,93 (m, 1H, Ar-H_6_); 7.91 (m, 1H, Ar-H_3_); 7.79 (d, *J* = 7.55 Hz, 1H, Ar-H_5_); 7.65 (d, *J* = 7.74 Hz, 1H, Ar-H_4_); 7.15 (d, 1H, *J*_(C-H)-(N-H)_ = 8.12 Hz, N-H); 6.91 (d, 1H, *J*_(C-H)-(O-H)_ = 5.33 Hz, O-H); 5.96 (dd, 1H, *J*_(C-H)-(N-H)_ = 8.12 Hz, *J*_(C-H)-(O-H)_ = 5.33 Hz, C-H); 2.27 (s, 6H, Tr-CH_3_.). ^13^C-NMR (DMSO-*d*_6_, 298 K, ppm, 75 MHz): δ = 151.6 (Tr-C), 148.9 (Ar-C_2_), 133.6 (Ar-C_1_), 133.4 (Ar-C_5_), 130.2 (Ar-C_4_), 129.1 (Ar-C_6_), 124.2 (Ar-C_3_), 79.2 (C-OH), 10.5 (Tr-CH_3_).

Crystal data (C_12_H_13_N_5_O_3_): M = 263.26, crystal system: monoclinic, space group: C2/c, a = 18.695(5) Å, b = 10.752(3) Å, c = 15.422(4) Å, β = 125.09(3)°, V = 2536.59(12) Å^3^, Z = 8, ρ_c_ = 1.379 g·cm^−3^, μ = 0.104 mm, θ_max_ = 29.43°, reflections: 5365, independent: 2885, R_int_ = 0.0155, R1 = 0.0458, wR2 = 0.1238, GoF = 1.026.

*(4H-3,5-Dimethyl-1,2,4-triazole-4-ylamino)(3-nitrophenyl)methanol* (**3**): Yield 48%. Anal. Calc. (%) for C_11_H_13_N_5_O_3_: C, 50.18; H, 4.98; N, 26.61. Found: C, 50.01; H, 4.46; N, 26.71. IR (KBr, cm^−1^): 503 w, 583 w, 608 w, 629 w, 679 s, 693 w, 730 m, 762 w, 803 m, 866 w, 907 w; 917 w; 944 w; 1003 w; 1050 s; 1093 m; 1202 m; 1248 w; 1353 vs; 1379 m; 1421 s; 1527 vs, 1563 m, 1585 w, 1617 w, 1648 w, 2931 m, 3115 s, 3253 s, 3312 s. Raman (cm^−1^): 186 vw, 234 vw, 348 vw, 420 vw, 608 vw, 632 vw, 679 vw, 681 vw, 726 vw, 764 vw, 861 vw, 1000 m, 1090 vw, 1160 vw, 1200 w, 1340 s, 1350 vs, 1440 vw. 1540 vw, 1560 vw, 1580 m, 1620 vw, 1690 vw, 2940 vw, 3090 vw, 3310 vw, 3320 vw. MS (ESI, *m*/*z*): 264.1 [M + H]^+^; 286.1 [M + Na]^+^; 302.1 [M + K]^+^, 549.2 [2M + Na]^+^. ^1^H-NMR (DMSO-*d*_6_, 298 K, ppm, 500 MHz): δ = 8.43 (s, 1H, Ar-H_2_); 8.26 (dd, 1H, *J*_4-5_ = 8.09 Hz,; *J*_4-6_ = 1.62 Hz, Ar-H_4_); 8.03 (d, 1H, *J*_5-6_ = 7.98 Hz, Ar-H_6_); 7.74 (t, 1H, *J* = 7.98 Hz, H_5_); 7.19 (d, 1H, *J*_(C-H)-(N-H)_ = 7.17 Hz, N-H); 6.79 (d, 1H, *J*_(C-H)-(O-H)_ = 5.78 Hz, O-H); 557 (t, 1H, *J*_(C-H)-(O-H),(N-H)_ = 6.47 Hz, C-H); 2.31 (s, 6H, Tr-CH_3_)). ^13^C-NMR (DMSO-*d*_6_, 298 K, ppm, 75 MHz): δ = 151.5 (Tr-C), 148.1 (Ar-C_3_), 143.0 (Ar-C_1_), 134.2 (Ar-C_6_), 130.2 (Ar-C_5_), 123.6 (Ar-C_4_), 121.8 (Ar-C_2_), 83.2 (C-OH), 10.7 (Tr-CH_3_).

*(4H-3,5-Dimethyl-1,2,4-triazole-4-ylamino)(4-nitrophenyl)methanol* (**4**): Yield 48%. Anal. Calc. (%) for C_11_H_13_N_5_O_3_: C, 50.18; H, 4.98; N, 26.61. Found: C, 50.26; H, 4.63; N, 26.50. IR (KBr, cm^−1^): 385 m, 477 m, 509 m, 528 w, 592 s, 628 w, 656 m, 678 m, 693 s, 718 s, 752 s, 766 m, 789 vs, 857 s, 896 s, 980 w, 1015 s, 1064 vs, 1106 s, 1119 w, 1191 s, 1250 s, 1273 s, 1350 vs, 1384 s, 1419 s, 1463 s, 1511 vs, 1569 vs, 1600 s, 1607 s, 2705 s, 2854 vs, 2914 vs, 3079 vs, 3304 vs. Raman (cm^−1^): 318 vw, 607 vw, 630 w, 679 vw, 765 vw, 859 vw, 896 vw, 1110 m, 1180 vw, 1340vs, 1520 vw, 1600 s, 2940 vw, 3070 vw, 3100 vw, 3310 vw. MS (ESI, *m*/*z*): 264.1 [M + H]^+^; 286.1 [M + Na]^+^; 302.1 [M + K]^+^. ^1^H-NMR (DMSO-*d*_6_, 298 K, ppm, 500 MHz): δ = 8.28 (d, 2H, *J*_3-2_ = 8.65 Hz, Ar-H_3,5_); 7.83 (d, 2H, *J*_2-3_ = 8.65 Hz, H_A_); 7.17 (d, 1H, *J*_(C-H)-(N-H)_ = 7.18 Hz, N-H); 6.76 (d, 1H, *J*_(C-H)-(O-H)_ = 5.67 Hz, O-H); 5,58 (t, 1H, *J*_(C-H)-(O-H),(N-H)_ = 6.42 Hz, C-H); 223 (s, 6H, Tr-CH_3_). ^13^C-NMR (DMSO-*d*_6_, 298 K, ppm, 75 MHz): δ = 151.5 (Tr-C), 147.9 (Ar-C_4_), 131.1 (Ar-C_1_), 128.7 (Ar-C_2,6_), 123.7 (Ar-C_3,5_), 83.4 (C-OH), 10.7 (Tr-CH_3_).

*(2,4-Dinitrophenyl)(4H-3,5-dimethyl-1,2,4-triazole-4-ylamino)methanol* (**5**): Yield 85%. Anal. Calc. (%) for C_11_H_12_N_6_O_5_: C, 42.86; H, 3.92; N, 27.26. Found: C, 42.76; H, 3.43; N, 27.00. IR (KBr, cm^−1^): 502 w, 588 w, 663 w, 678 vw, 712 m, 727 w, 743 w, 760 w, 766 w, 790 m, 835 m, 889 w, 905 m, 1022 w, 1059 m, 1083 m, 1127 w, 1147 vw, 1191 w, 1247 w, 1295 w, 1350 vs, 1415 m, 1469 w, 1506 m, 1537 vs, 1566 m, 1608 m, 2744 m, 2879 m, 3106 m, 3305 s. Raman (cm^−1^): 265 vw, 281 vw, 318 vw, 346 w, 610 vw, 650 w, 680 vw, 695 vw, 699 w, 715 vw, 836 m, 889 vw, 923 vw, 1020 vw, 1060 vw, 1130 w, 1150 m, 1190 vw, 1350 vs 1370 s, 1410 vw, 1460 vw, 1550 w, 1610 vs, 2950 vw, 2990 vw, 3010 vw, 3090 vw, 3110 vw, 3310 vw. MS (ESI, *m*/*z*): 301.1 [M + H]^+^; 331.1 [M + Na]^+^; 347.1 [M + K]^+^. ^1^H-NMR (DMSO-*d*_6_, 298 K, ppm, 500 MHz): δ = 8.76 (d, 1H, *J*_3-5_ = 2.30 Hz, Ar-H_3_); 8.62 (dd, 1H, *J*_5-6_ = 8.77 Hz, *J*_3-5_ = 2.30 Hz, Ar-H_5_); 8.21 (d, 1H, *J*_6-5_ = 8.77 Hz, Ar-H_6_); 7.33 (d, 1H, *J*_(C-H)-(N-H)_ = 8.35 Hz, N-H); 7.26 (d, 1H, *J*_(C-H)-(O-H)_ = 5.01 Hz, O-H); 6.00 (dd, 1H, *J*_(C-H)-(N-H)_ = 8.35 Hz, *J*_(C-H)-(O-H)_ = 5.01 Hz, C-H); 2.23 (s, 6H, Tr-CH_3_). ^13^C-NMR (DMSO-*d*_6_, 298 K, ppm, 75 MHz): δ = 151.5 (Tr-C), 148.7 (Ar-C_2_), 147.9 (Ar-C_4_), 139.7 (Ar-C_1_), 131.0 (Ar-C_5_), 127.6 (Ar-C_6_), 119.8 (Ar-C_3_), 79.1 (C-OH), 10.7 (Tr-CH_3_).

Crystal data: (C_11_H_12_N_6_O_5_∙H_2_O) M = 326.28, crystal system: triclinic, space group: *P* $\overset{´}{1}$, a = 8.054(3) Å, b = 8.078(3) Å, c = 12.459(3) Å, α = 87.60(3)°, β = 81.60(3)°, γ = 66.48(3)°, V = 735.1(4) Å^3^, Z = 2, ρ_c_ = 1.474 g·cm^−3^, μ = 0.122 mm, θ_max_ = 28.77°, reflections: 12648, independent: 3517, R_int_ = 0.0393, R1 = 0.0595, wR2 = 0.1553, GoF = 1.035.

*(4-Chloro-3-nitrophenyl)(4H-3,5-dimethyl-1,2,4-triazole-4-ylamino)methanol* (**6**): Yield 46%. Anal. Calc. (%) for C_11_H_12_ClN_5_O_3_: C 44.38; H, 4.06; N, 23.53. Found: C, 44.35; H, 3.56; N, 23.31. IR (KBr, cm^−1^): 487 w, 505 w, 586 w, 603 w, 633 w, 664 w, 680 w, 691 w, 744 w, 763 w, 809 m, 863 m, 917 vw, 947 w, 980 vw, 1025 m, 1048 s, 1091 w, 1138 w, 1204 w, 1249 w, 1367 s, 1423 m, 1479 w, 1502 m, 1532 vs, 1566 m, 1607 w, 2729 w, 2878 m, 3105 m, 3261 s, Raman (cm^−1^): 272 vw, 330 w, 409 vw, 487 vw, 490 vw, 515 vw, 607 vw, 637 vw, 667 vw, 711 vw, 763 vw, 812 vw, 861 vw, 1050 w, 1140 w, 1160 w, 1200 w, 1230 w, 1290 vw, 1370 w, 1540 w, 1560 m, 1610 vs, 2940 vw, 2980 vw, 3000 vw, 3010 vw, 3270 vw. MS (ESI, *m*/*z*): 298.1 [M + H]^+^, 320.0 [M + Na]^+^, 336.0 [M + K]^+^. ^1^H-NMR (DMSO-*d*_6_, 298 K, ppm, 500 MHz): δ = 8.23 (d, 1H, *J*_2-6_ = 1.72 Hz, Ar-H_2_); 7.89 (d, 1H, *J*_2-6_ = 1.72 Hz, Ar-H_6_); 7.87 (m, 1H, Ar-H_5_); 7.20 (d, 1H, *J*_(C-H)-(N-H)_ = 7.44 Hz, N-H); 6.86 (d, 1H, *J*_(C-H)-(O-H)_ = 5.72 Hz, O-H); 5.57 (t, 1H, *J*_(C-H)-(O-H),(N-H)_ = 6.58 Hz, C-H); 2.36 (s, 6H, Tr-CH_3_). ^13^C-NMR (DMSO-*d*_6_, 298 K, ppm, 75 MHz): δ = 151.5 (Tr-C), 147.8 (Ar-C_3_), 141.8 (Ar-C_1_), 132.8 (Ar-C_6_), 131.9 (Ar-C_5_), 125.0 (Ar-C_4_), 124.2 (Ar-C_2_), 82.7 (C-OH), 10.7 (Tr-CH_3_).

*(4H-3,5-Dimethyl-1,2,4-triazole-4-ylamino)(4-methyl-3-nitrophenyl)methanol* (**7**): Yield 53%. Anal. Calc. (%) for C_12_H_15_N_5_O_3_: C 51.98; H, 5.45; N, 25.26. Found: C, 51.99; H, 5.05; N, 24.96. IR (KBr, cm^−1^): 504 w, 540 vw, 569 m, 615 w, 666 w, 682 m, 731 m, 754 m, 762 w, 777 m, 810 m, 862 m, 912 w, 952 w, 1024 w, 1065 s, 1080 s, 1165 vw, 1198 m, 1246 w, 1331 vs, 1346 vs, 1382 m, 1419 m, 1454 m, 1500 s, 1524 vs, 1564 m, 1572 s, 1623 w, 2723 m, 2873 m, 2924 m, 2991 m, 3105 s, 3309 vs. Raman (cm^−1^): 190 vw, 282 vw, 340 w, 394 vw, 427 vw, 572 vw, 616 w, 668 vw, 682 vw, 731 vw, 810 w, 862 vw, 952 vw, 1010 vw, 1070 vw, 1200 m, 1330 vs, 1390 w, 1450 vw, 1530 w, 1540 vw, 1570 w, 1630 vw, 2940 w, 2990 vw, 3060 vw, 3070 vw, 3100 vw, 3310 vw. MS (ESI, *m*/*z*): 278.1 [M + H]^+^, 300.1 [M + Na]^+^, 316.1 [M + K]^+^. ^1^H-NMR (DMSO-*d*_6_, 298 K, ppm, 500 MHz): δ = 8.16 (d, 1H, *J*_2-6_ = 1.18 Hz, Ar-H_2_); 7.76 (dd, 1H, *J*_6-5_ = 7.82 Hz, *J*_6-2_ = 1.42 Hz, Ar-H_6_); 7.58 (d, 1H, J_B-C_ = 7.82 Hz, Ar-H_5_); 7.12 (d, 1H, *J*_(C-H)-(N-H)_ = 6.87 Hz, N-H); 6.70 (d, 1H, *J*_(C-H)-(O-H)_ = 5.69 Hz, O-H); 5.54 (t, 1H, *J*_(C-H)-(O-H),(N-H)_ = 6.40 Hz, C-H); 2.56 (s, 3H, Ar-CH_3_); 2,35 (s, 6H, Tr-CH_3_)). ^13^C-NMR (DMSO-*d*_6_, 298 K, ppm, 75 MHz): δ = 151.0 (Tr-C), 148.6 (Ar-C_3_), 139.9 (Ar-C_1_,_4_) 132.6 (Ar-C_5_), 131.7 (Ar-C_6_), 122.4 (Ar-C_2_), 82.6 (C-OH), 19.3 (Ar-CH_3_), 10.2 (Tr-CH_3_).

*(2-Chloro-5-nitrophenyl)(4H-3,5-dimethyl-1,2,4-triazole-4-ylamino)methanol* (**8**): Yield 74%. Anal. Calc. (%) for C_11_H_12_ClN_5_O_3_: C 44.38; H, 4.06; N, 23.53; Cl, 11.91. Found: C, 44.39; H, 3.65; N, 23.21; Cl, 12.15. IR (KBr, cm^−1^): 465 w, 502 w, 511 w, 528 m, 570 w, 585 m, 612 m, 630 w, 662 m, 682 w, 692 w, 745 s, 769 w, 800 m, 842 m, 858 w, 913 m, 950 w, 985 w, 1027 m, 1041 s, 1068 m, 1103 m, 1196 m, 1247 m, 1278 m 1351 vs, 1377 m, 1419 s, 1462 m, 1517 vs, 1550 m, 1576 m, 1610 m, 2858 m, 3070 s, 3099 s, 3309 s. Raman (cm^−1^): 240 vw, 278 vw, 318 vw, 340 vw, 427 vw, 502 vw, 597 vw, 631 vw, 666 vw, 696 vw, 725 vw, 768 vw, 801 vw, 819 vw, 858 vw, 949 vw, 985 vw, 1024 vw, 1067 vw, 1103 vw, 1196 vw, 1348 vs, 1518 vw, 1544 vw, 1575 w, 1609 vw, 1703 vw, 2942 vw, 3081 vw, 3309 vw. MS (ESI, *m*/*z*): 298.1 [M + H]^+^, 320.0 [M + Na]^+^, 336.0 [M + K]^+^. ^1^H-NMR (DMSO-*d*_6_, 298 K, ppm, 500 MHz): δ = 8.50 (d, 1H, *J*_6-4_ = 2.83 Hz, Ar-H_6_); 8.27 (dd, 1H, *J*_4-3_ = 8.88 Hz, *J*_4-6_ = 2.83 Hz, Ar-H_4_); 7.84 (d, 1H, *J*_3-4_ = 8.88 Hz, Ar-H_3_); 7.26 (d, 1H, *J*_(C-H)-(N-H)_ = 6.99 Hz, N-H); 7.02 (d, 1H, *J*_(C-H)-(O-H)_ = 5.48 Hz, O-H); 5.77 (dd, 1H, *J*_(C-H)-(N-H)_ = 6.99 Hz, *J*_(C-H)-(O-H)_ = 5.48 Hz, C-H); 2.36 (s, 6H, Tr-CH_3_). ^13^C-NMR (DMSO-*d*_6_, 298 K, ppm, 125 MHz): δ = 151.1 (Tr-C), 146.4 (Ar-C_5_), 139.3 (Ar-C_2_), 138.5 (Ar-C_1_), 130.9 (Ar-C_3_), 124.8 (Ar-C_4_), 123.4 (Ar-C_6_) 80.0 (C-OH), 10.3 (Tr-CH_3_).

*(2-Chlorophenyl)(4H-3,5-dimethyl-1,2,4-triazole-4-ylamino)methanol* (**9**): Yield 63%. Anal. Calc. (%) for C_11_H_13_ClN_4_O: C 52.28; H, 5.19; N, 22.17. Found: C, 52.36; H, 4.94; N, 21.95. IR (KBr, cm^−1^): 428 w, 464 s, 509 m, 588 s, 613 m, 633 s, 667 s, 677 m, 705 s, 742 vs, 759 vs, 805 m, 884s, 955 w, 978 w, 994 m, 1016 vs, 1035 s, 1047 s, 1057 s, 1088 m, 1196 m, 1247 w, 1265 w, 1340 w, 1357 w, 1374 m, 1419 s, 1440 s, 1471 s, 1507 m, 1542 m, 1567 m, 1578 m, 1598 w, 2880 m, 2033 m, 3124 m, 3265 s. Raman (cm^−1^): 179 vw, 218 w, 259 w, 280 w, 323 w, 330 w, 354 w, 431 vs, 590 w, 615 m, 625 m, 634 w, 670 w, 681 w, 705 w, 739 vw, 764 w, 882 w, 997 m, 1037 vs, 1085 w, 1127 vw, 1159 s, 1196 m, 1212 vw, 1274 w, 1287 w, 1318 vw, 1378 w, 1435 w, 1468 w, 1543 vs, 1576 vs, 1592 m, 1598 m, 1611 w, 1696 vw, 2932 m, 2991 vw, 3059 m, 3070 m, 3081 m, 3265 vw. MS (ESI, *m*/*z*): 253.1 [M + H]^+^, 505.2 [2M + H]^+^. ^1^H-NMR (DMSO-*d*_6_, 298 K, ppm, 500 MHz): δ = 7.67 (m, 1H, Ar-H_3_), 7.48 (m, 1H, Ar-H_6_), 7.41 (m, 2H, Ar-H_4_,H_5_), 7.05 (d, 1H, *J*_(C-H)-(N-H)_ = 6.29 Hz, N-H), 6.60 (d, 1H, *J*_(C-H)-(O-H)_ = 5.15 Hz, O-H), 5.76 (t, 1H, *J*_(C-H)-(O-H),(N-H)_ = 5.72 Hz, C-H), 2.30 (s, 6H, Tr-CH_3_). ^13^C-NMR (DMSO-*d*_6_, 298 K, ppm, 75 MHz): δ = 151.8 (Tr-C), 137.9 (Ar-C_1_), 132.0 (Ar-C_2_), 130.6 (Ar-C_4_), 129.6 (Ar-C_6_), 129.1 (Ar-C_3_), 127.7 (Ar-C_5_), 80.8 (C-OH), 10.7 (Tr-CH_3_).

Crystal data: M = 252.70, crystal system: monoclinic, space group: *P2_1_*/*c*, a = 10.882(3) Å, b = 14.734(4) Å, c = 7.420(3) Å, β = 104.5(3)°, V = 1151.7(6) Å^3^, Z = 4, ρ_c_ = 1.457 g·cm^−3^, μ = 0.321 mm, θ_max_ = 28.66°, reflections: 8449, independent: 2788, R_int_ = 0.0228, R1 = 0.1481, wR2 = 0.08908, GoF = 0.974.

*(4H-3,5-Dimethyl-1,2,4-triazole-4-ylamino)(4-formylphenyl)methanol* (**10**): Yield 39%. Anal. Calc. (%) for C_12_H_14_N_4_O_2_: C, 58.53; H, 5.73; N, 22.75. Found: C, 58.42; H, 5.41; N, 22.28. IR (KBr, cm^−1^): IR (KBr, cm^−1^): 490 w, 599 m, 699 m, 706 w, 760 s, 782 s, 843 m, 893 m, 981 w, 1016 m, 1026 m, 1064 vs, 1113 w, 1166 m, 1210 s, 1252 w, 1273 m, 1304 m, 1335 w, 1384 s, 1419 s, 1509 m, 1541 m, 1568 s, 1608 s, 1700 vs, 2716 s, 2819 s, 2915 m, 3077 s, 3291vs. Raman (cm^−1^): 203 vw, 280 vw, 306 vw, 332 vw, 600 vw, 631 vw, 643 vw, 671 vw, 706 vw842 vw, 892 vw, 1021 vw, 1062 vw, 1112 vw, 1171 w, 1210 vw, 1380 vw, 1466 vw, 1510 vw, 1544 vw, 1560 vw, 1580 vw, 1607 vs, 1695 m, 2233 vw, 2731 vw, 2929 vw, 2970 vw, 2997 vw, 3064 vw, 3080 vw, 3293 vw. MS (ESI, *m*/*z*): 269.1 [M + H]^+^. ^1^H-NMR (DMSO-*d*_6_, 298 K, ppm, 500 MHz): δ = 7.96 (d, 2H, *J*_2-3_ = 8.20 Hz, Ar-H_3,5_), 7.79 (d, 2H, *J*_2-5_ = 8.20 Hz, Ar-H_2,6_), 7.12 (d, 1H, *J*_(C-H)-(N-H)_ = 6.87 Hz, N-H), 6.64 (d, 1H, *J*_(C-H)-(O-H)_ = 5.53 Hz, O-H), 5.55 (t, 1H, *J*_(C-H)-(O-H),(N-H)_ = 6.20 Hz, C-H), 2.34 (s, 6H, Tr-CH_3_). ^13^C-NMR (DMSO-*d*_6_, 298 K, ppm, 125 MHz): δ = 193.4 (Ar-CHO), 151.6 (Tr-C), 147.1 (Ar-C_3,5_), 136.5 (Ar-C_4_), 129.8 (Ar-C_2,6_), 128.0 (Ar-C_1_), 83.9 (C-OH), 10.8 (Tr-CH_3_).

Crystal data: M = 246.27, crystal system: monoclinic, space group: *P2*_1_/*c*, a = 12.759(4) Å, b = 7.395(3) Å, c = 12.768(6) Å,, β = 96.91(4)°, V = 1195.81(8) Å^3^, Z = 4, ρ_c_ = 1.368 g·cm^−3^, μ = 0.097 mm, θ_max_ = 24.99°, reflections: 12521, independent: 2113, R_int_ = 0.0423, R1 = 0.0424, wR2 = 0.111, GoF = 1.000.

*(4-Cyanophenyl)(4H-3,5-dimethyl-1,2,4-triazole-4-ylamino)methanol* (**11**): Yield 51%. Anal. Calc. (%) for C_12_H_13_N_4_O: C 59.25; H, 5.39; N, 28.79. Found: C, 59.23; H, 5.03; N, 28.40. IR (KBr, cm^−1^): 461 w, 481 vw, 503 w, 549 m, 603 s, 664 m, 712 w, 751 s, 768 w, 802 m, 817 m, 837 m, 894 m, 1019 m, 1064 vs, 1198 w, 1249 w, 1272 w, 1333 w, 1352 w, 1385 m, 1408 m, 1457 m, 1544 w, 1569 m, 1610 w, 2233 s, 2704 w, 2855 w, 2914 w, 3082 m, 3280 s. Raman (cm^−1^): 315 vw, 491 vw, 558 vw, 608 vw, 646 vw, 718 vw, 747 vw, 818 vw, 880 vw, 1010 m, 1186 w, 1223 w, 1290 vw, 1319 vw, 1364 vw, 1411 vw, 1508 vw, 1538 vs, 1581 vs, 1610 s, 2227 m, 2928 vw, 2988 vw, 3051 vw, 3084 vw, 3281 vw. MS (ESI, *m*/*z*): 244.1 [M + H]^+^, 266.1 [M + Na]^+^, 509.2 [2M + Na]^+^. ^1^H-NMR (DMSO-*d*_6_, 298 K, ppm, 500 MHz): δ = 7.89 (d, 2H, *J*_2-3_ = 8.20 Hz, Ar-H_3,5_), 7.75 (m, 2H, *J*_2-3_ = 8.20 Hz, Ar-H_2,6_), 7.13 (d, 1H, *J*_(C-H)-(N-H)_ = 7.06 Hz, N-H), 6.70 (d, 1H, *J*_(C-H)-(O-H)_ = 5.72 Hz, O-H), 5.53 (t, 1H, *J*_(C-H)-(O-H),(N-H)_ = 6.49 Hz, C-H), 2.34 (s, 6H, Tr-CH_3_)). ^13^C-NMR (DMSO-*d*_6_, 298 K, ppm, 125 MHz): δ = 151.7 (Tr-C), 147.0 (Ar-C_1_), 132.6 (Ar-C_3,5_), 128.3 (Ar-C_2,6_), 119.2 (Ar-CN), 111.4 (Ar-C_4_), 83.6 (C-OH), 10.7 (Tr-CH_3_).

*(4H-3,5-Dimethyl-1,2,4-triazole-4-ylamino)(4-(trifluoromethyl)phenyl)methanol* (**12**): Yield 39%. Anal. Calc. (%) for C_12_H_13_F_3_N_4_O: C 50.35; H, 4.58; N, 19.57. Found: C, 50.3; H, 5.03; N, 18.87. IR (KBr, cm^−1^): 461 w,502 w,581 w, 597 m, 654 w, 668 w, 712 w, 754 m, 770 m, 810 m, 829 m, 896 m, 1016 s, 1053 vs, 1070 vs, 1111 vs, 1152 vs, 1205 m, 1247 w, 1283 w, 1332 vs, 1380 m, 1415 s, 1498 m, 1542 m, 1565 s, 1622 w, 2729 w, 2894 m, 2933 m, 3126 s, 3284 s. Raman (cm^−1^): 228 s, 275 w, 299 w, 315 m, 342 w, 400 vw, 415 w, 500 w, 579 w, 604 s, 635 vs, 655 w, 670 s, 679 w, 710 m, 740 m, 753 m, 768 m, 801 w, 894 m, 980 w, 1014 vw, 1050 vw, 1067 w, 1081 vw, 1104 vw, 1185 m, 1204 m, 1284 vw, 1324 vs, 1361 vw, 1384 w, 1463 w, 1540 m, 1592 w, 1621 vs, 2739 vw, 2932 s, 2991 w, 3011 w, 3082 s, 3285 vw. MS (ESI, *m*/*z*): 287.1 [M + H]^+^, 309.1 [M + Na]^+^, 325.1 [M + K]^+^. ^1^H-NMR (DMSO-*d*_6_, 298 K, ppm, 500 MHz): δ = 7.79 (s, 4H, Ar-H_2,3,5,6_), 7.11 (d, 1H, *J*_(C-H)-(N-H)_ = 6.87 Hz, N-H), 6.66 (d, 1H, *J*_(C-H)-(O-H)_ = 5.53 Hz, O-H), 5.55 (t, 1H, *J*_(C-H)-(O-H),(N-H)_ = 6.20 Hz, C-H), 2.34 (s, 6H, Tr-CH_3_)). ^13^C-NMR (DMSO-*d*_6_, 298 K, ppm, 125 MHz): δ = 151.6 (Tr-C), 145.3 (Ar-C_1_), 129.3 (q, ^2^*J*_F-C_ = 30.88 Hz, Ar-C_4_), za 128.2 (Ar-C_2,6_), 125.5 (q, ^3^*J*_F-C_ = 3.63 Hz, Ar-C_3,5_), 124.7 (q, ^1^*J*_F-C_ = 272.4 Hz, Ar-CF_3_), 83.7 (C-OH), 10.7 (Tr-CH_3_).

Compounds **13** and **14** were synthesized and purified according to the following procedure. A solution of suitable pyridinecarboxaldehyde (0.47 mL, 0.5 mM) in 1 mL of ethanol was added to hot solution of equimolar amounts of MeATR (**1**) (0.56 g, 0.5 mM) in 10 mL of ethanol. The reaction mixture was refluxed for 2 h, cooled and kept overnight in refrigerator. The solvent was then removed *in vacuo* and the remaining materials were washed with cold ethanol and dried in air.

*(4H-3,5-Dimethyl-1,2,4-triazole-4-ylamino)(pyridin-3-yl)methanol* (**13**): Yield 84%. Anal. Calc. (%) for C_10_H_13_N_5_O: C, 54.78; H, 5.98; N, 31.94. Found: C, 54.80; H, 6.12; N, 32.18. IR (nujol, cm^−1^): 410 w, 490 w, 596 w, 613 w, 622 w, 651 w, 675 w, 707 s, 746 w, 766 m, 784 m, 831 w, 894 m, 955 m, 980 w, 1030 m, 1051 m, 1065 vs, 1086 m, 1205 w, 1252 w, 1300 w, 1336 m, 1353 w, 1425 vs, 1511 m, 1525 m, 1542 m, 1570 s, 1582 m, 1596 m, 2741 m, 3069 s, 3125 s, 3202 s. Raman (cm^−1^): 225 vw, 265 vw, 294 vw,. 311 vw, 338 vw, 357 vw, 409 vw, 487 vw, 597 w, 622 w, 653 vw, 677 vw, 743 vw, 764 vw, 799 vw, 832 vw, 891 vw, 980 vw, 1028 w, 1041 vs, 1062 vw, 1085 vw, 1127 vw, 1191 w, 1254 vw, 1300 vw, 1335 vw, 1380 vw, 1457 vw, 1523 vw, 1541 w, 1572 vw, 1596 w, 1618 w, 2930 m, 2984 vw, 3054 w, 3062 w, 3078 w, 3203 vw. MS (ESI, *m*/*z*): 220.1 [M + H]^+^, 242.1 [M + Na]^+^, 258.1 [M + K]^+^, 461.2 [2M + Na]. ^1^H-NMR (DMSO-*d*_6_, 298 K, ppm, 500 MHz): δ = 8.86 (d, 1H, *J*_2-6_ =1.89 Hz, Py-H_2_), 8.57 (dd, 1H, *J*_4-5_ = 4.82 Hz,, *J*_4-6_ = 1.61 Hz Py-H_4_), 7.93 (dt, 1H, *J*_5-6_ = 7.90 Hz, *J*_2,4-6_ = 1.70 Hz), 7.45 (ddd, 1H, *J*_5-6_ = 7.90 Hz, *J*_4-5_ = 4.82 Hz, *J*_2-5_ = 0.66 Hz, Py-H_5_), 7.13 (d, 1H, *J*_(C-H)-(N-H)_ = 6.99 Hz, N-H), 6.66 (d, 1H, *J*_(C-H)-(O-H)_ = 5.67 Hz, O-H), 5.54 (t, 1H, *J*_(C-H)-(O-H),(N-H)_ = 6.33 Hz, C-H), 2.34 (s, 6H, Tr-CH_3_). ^13^C-NMR (DMSO-*d*_6_, 298 K, ppm, 125 MHz): δ = 151.1 (Tr-C), 149.4 (Py-C_4_), 148.2 (Py-C_2_), 139.2 (Py-C_1_), 134.2 (Py-C_6_), 123.3 (Py-C_5_), 82.2 (C-OH), 10.3 (Tr-CH_3_).

*(4H-3,5-Dimethyl-1,2,4-triazole-4-ylamino)(pyridin-4-yl)methanol* (**14**): Yield 81%. Anal. Calc. (%) for C_10_H_13_N_5_O: C, 54.78; H, 5.98; N, 31.94. Found: C, 54.79; H, 6.06; N, 32.51. IR (nujol, cm^−1^): 406 w, 503 w, 582 m, 611 w, 637 m, 671 m, 764 m, 779 m, 803 s, 848 w, 903 s, 977 w, 997 m, 1031 m, 1061 vs, 1105 s, 1197 m, 1216 w, 1232 w, 1245 w, 1289 w, 1320 w, 1339 m, 1415 vs, 1512 m, 1541 m, 1573 s, 1609 m, 2724 m, 3061 s, 3122 s, 3191s. Raman (cm^−1^): 226 vw, 269 vw, 294 vw, 313 vw, 336 vw, 352 vw, 405 vw, 500 vw, 523 vw, 591 vw, 612 vw, 636 w, 670 s, 680 vw, 725 w, 762 w, 805 vw, 851 vw, 901 vw, 1001 vs, 1029 vw, 1058 vw, 1094 vw, 1192 w, 1214 vw, 1228 vw, 1287 vw, 1320 vw, 1335 vw, 1380 vw, 1450 vw, 1540 w, 1562 vw, 1607 w, 1620 w, 2739 vw, 2931 w, 2975 vw, 3059 w, 3108 vw, 3196 vw. MS (ESI, *m*/*z*): 220.1 [M + H]^+^, 242.1 [M + Na]^+^, 258.1 [M + K]^+^. ^1^H-NMR (DMSO-*d*_6_, 298 K, ppm, 500 MHz): δ = 8.62 (d, 1H, *J*_2-3_ = 5.85 Hz, Py-H_3,5_), 7.56 (d, 1H, *J* = 5.67 Hz, Py-H_2,6_), 7.15 (d, 1H, *J*_(C-H)-(N-H)_ = 7.18 Hz, N-H), 6.69 (d, 1H, *J*_(C-H)-(O-H)_ = 5.85 Hz, O-H), 5.47 (t, 1H, *J*_(C-H)-(O-H),(N-H)_ = 6.61 Hz, C-H), 2.34 (s, 6H, Tr-CH_3_). ^13^C-NMR (DMSO-*d*_6_, 298 K, ppm, 125 MHz): δ = 151.5 (Tr-C), 150.1 Py-C_3,5_), 149.0 (Py-C_1_), 122.3 (Py-C_2_,_6_), 83.2 (C-OH), 10.7 (Tr-CH_3_).

### 3.4. Synthesis of Imines **15**–**16**

Schiff basses were prepared according to the following general procedure. A mixture of equimolar amounts (0.5 mmol) of MeATR (**1**) and an appropriate aldehyde ArCHO (in molar ratio 1:1) were dissolved in acetonitrile (3 mL) with presence of catalytic amounts of hydrochloric acid (2 drops, 36%,). The reaction mixture was then refluxed for 3 h. After cooling, the precipitate formed was filtered off, washed with small amount of cold acetonitrile and then dried in the air.

*(N-(4-Nitrobenzylidene)-4H-3,5-dimethyl-1,2,4-triazole-4-amine) hydrochloride* (**15**): Yield 97%. Anal. Calc. (%) for C_11_H_12_N_5_O_2_Cl: C, 46.90; H, 4.29; N, 24.86. Found: C, 46.99; H, 4.12; N, 24.52. IR (KBr, cm^−1^): 438 w; 503 w; 692 m; 757 m; 873 m; 884 m; 931 w; 980 w; 1006 m; 1018 m; 1045 m; 1113 m; 1201 vw; 1235 w; 1317 s; 1346 vs; 1376 m; 1405 m; 1475 w; 1489 w; 1525 vs; 1567 w; 1589 s; 1618 w; 1827 m; 2367 s; 2926 w; 2982 w; 3062 w. Raman (cm^−1^): 280 vw; 318 vw; 341 vw; 607 vw; 629 w; 678 vw; 725 vw; 765 vw; 859 vw; 897 vw; 1070 vw; 1110 m; 1180 vw; 1200 vw; 1340vs; 1520 vw; 1560 vw; 1600 s; 1620 vw; 1710 vw; 2940 vw. MS (ESI, *m*/*z*, M = C_11_H_11_N_5_O_2_): 246.1 [M + H]^+^, 268.1 [M + Na]^+^, 284.1 [M + K]^+^. 387.6 [(C_11_H_11_N_5_O)_3_(HCl)_2_O]^2+^, 404.6 [(C_11_H_12_N_5_O_2_)_3_(HCl)_2_]^2+^. ^1^H-NMR (DMSO-*d*_6_, 298 K, ppm, 300 MHz): δ = 9.17 (s, 1H, CH=N); 8.40 (d, 2H, *J*_2-3_ = 9.00 Hz, Ar-H_3,5_); 8.23 (d, 2H, *J*_2-3_ = 9.00 Hz, Ar-H_2,6_); 2.59 (s, 6H, Tr-CH_3_). ^13^C-NMR (DMSO-*d*_6_, 298 K, ppm, 75 MHz): δ = 166.9 (CH=N), 150.5 (Ar-C_4_), 149.1 (Tr C), 137.6 (Ar-C_1_), 131.0 (Ar-C_2,6_), 124.7 (Ar-C_3,5_), 10.8 (Tr-CH_3_).

Crystal data (C_11_H_11_N_5_O_2_∙HCl): M = 343.73, crystal system: triclinic, space group: *P* $\overset{´}{1}$, a = 7.349(3) Å, b = 9.537(3) Å, c = 9.812(3) Å, α = 84.87(3)°, β = 76.62(3)°, γ = 88.19(3)°, V = 666.3(4) Å^3^, Z = 2, ρ_c_ = 1.713 g·cm^−3^, μ = 0.326 mm, θ_max_ = 28.75°, reflections: 4597, independent: 3002, R_int_ = 0.0130, R1 = 0.0393, wR2 = 0.1016, GoF = 1.023.

*(N-(4-chloro-3-nitrobenzylidene)-4H-3,5-Dimethyl-1,2,4-triazole-4-amine) hydrochloride* (**16**): Yield 98%. Anal. Calc. (%) for C_11_H_12_N_5_O_2_Cl: C, 46.90; H, 4.29; N, 24.86. Found: C, 46.99; H, 4.12; N, 24.52. IR (KBr, cm^−1^): 422 w; 438 m; 503 m; 537 w; 545 w; 599 w; 649 w; 662 w; 671 w; 692 vs; 757 vs; 776 m; 846 vs; 873 vs; 884 s; 931 s; 980 s; 1006 vs; 1017 s; 1045 s; 1106 s; 1201 m; 1234 s; 1317 vs; 1346 vs; 1376 s; 1404 s; 1475 m; 1489 m; 1525 vs; 1567 m; 1589 s; 1618 m; 1824 s; 2351 s; 2926 w; 2983 w; 3062 w. MS (ESI, *m*/*z*, M = C_11_H_10_N_5_O_2_Cl): 280.1 [M + H]^+^, 302.0 [M + Na]^+^, 439.6 [3M∙HCl + 4H]^2+^, 581.1 [2M + Na]^+^. ^1^H-NMR (DMSO-*d*_6_, 298 K, ppm, 300 MHz): δ = 9.16 (s, 1H, CH=N); 8.69 (d, 1H, *J*_2-6_ = 1.90 Hz, Ar-H_2_); 8.29 (dd, 1H, *J*_5-6_ = 8.53 Hz, *J*_2-6_ = 1.90 Hz, Ar-H_6_); 8.01 (d, 1H, *J*_5-6_ = 8.53 Hz, Ar-H_5_); 2.60 (s, 6H, Tr-CH_3_). ^13^C-NMR (DMSO-*d*_6_, 298 K, ppm, 75 MHz): δ = 166.2 (CH=N), 149.2 (Tr C), 148.3 (Ar-C_3_), 134.1 (Ar-C_6_), 133.3 (Ar-C_5_), 132.3 (Ar-C_4_), 130.1 9Ar-C_1_), 126,7 (Ar-C_2_), 10.8 (Tr-CH_3_).

### 3.5. Reaction of MeATR *(**1**)* with 2-Pyridinecarboxaldehyde

A mixture of equimolar amounts (0.17 mmol) of MeATR (**1**) and 2-pyridinecarboxaldehyde (in molar ratio 1:1) were dissolved in hexane (2 mL) and stirred at 50 °C for 9 h. After removing volatile components, raw solid products washed with cold hexane dried and dissolved in DMSO-*d*_6_ and analyzed by NMR spectroscopy.

*(4H-3,5-Dimethyl-1,2,4-triazole-4-ylamino)(pyridin-2-yl)methanol* (**17**) Yield 12%.^1^H-NMR (DMSO-*d*_6_, 298 K, ppm, 500 MHz): δ = 8.62 (m, 1H, Py-H_3_), 7.89 (td, 1H, *J*_4-5,5-6_ = 7.68 Hz, *J*_3-5_ = 1.81 Hz Py-H_5_), 7.59 (d, 1H, *J*_5-6_ = 7.90 Hz, Py-H_6_), 7.41 (ddd, 1H, *J*_4-5_ = 7.44 Hz, *J*_3-4_ = 4.77 Hz, *J*_4-6_ = 1.14 Hz, Py-H_4_), 7.14 (d, 1H, *J*_(C-H)-(N-H)_ = 4.96 Hz, N-H), 6. 51 (d, 1H, *J*_(C-H)-(O-H)_ = 6.29 Hz, O-H), 5.44 (dd, 1H, *J*_(C-H)-(N-H)_ = 4.96 Hz, *J*_(C-H)-(O-H)_ = 6.29 Hz, C-H), 2.30 (s, 6H, Tr-CH_3_).

*(N-(Pyridin-2-yl,methylene)-4H-3,5-dimethyl-1,2,4-triazole-4-amine)* (**17a**) Yield 23%. ^1^H-NMR (DMSO-*d*_6_, 298 K, ppm, 500 MHz): δ = 8.81 (s, 1H), 8.77 (m, 1H, Py-H_3_), 8.17 (dt, 1H, *J*_5-6_ = 7.87 Hz, *J*_3,4-6_ = 1.03 Hz, Py-H_6_) 8.03 (m, 1H, Py-H_5_), 7.62 (ddd, 1H, *J*_4-5_ = 7.49 Hz, *J*_3-4_ = 4.82 Hz, *J*_4-6_ = 1.24 Hz, Py-H_4_), 2.47 (s, 6H, Tr-CH_3_).

### 3.6. Reaction Survey

The effect of substituents on the condensation reaction—aldehyde (0.172 mmol) and amine **1** (0.172 mmol) were dissolved in acetonitrile (2 mL) and stirred for 9 h at 50 °C. After removing volatile components, the solid products were dissolved in DMSO-*d*_6_ and ^1^H-NMR spectra were measured. The amount of hemiaminal, Schiff base and unreacted amine were determined from integrated peak intensities.

The effect of solvent on the condensation reaction—2-nitrobenzaldehyde (0.172 mmol), amine **1** (0.172 mmol) and 2 mL of solvent were stirred for 9 h at 50 °C. After removing volatile components, the solid products were dissolved in DMSO-*d*_6_ and ^1^H-NMR spectra were measured. The amount of hemiaminal (HA), Schiff base (SB) and unreacted amine (A) were determined from integrated peak intensities of the δ(C-CH_3_) signals (A—2.25 ppm, HA—2.27 ppm, SB—2.47 ppm).

## 4. Conclusions

In this paper, a new group of hemiaminals derived from aromatic aldehydes (benzyl, pyridyl) and 4-amine-3,5-dimethyl-1,2,4-triazole was presented. We found that most of the electron-withdrawing substituents in the aromatic aldehydes can stabilize the creation of stable hemiaminals e.g., compounds **9**, **10**, **12**, **13** and **14** presented in this paper. The presence of two methyl substituents in the triazole ring significantly affects the crystal and molecular structure of hemiaminals, which form centrosymmetric dimers only, while predominantly polymeric structures have been reported previously. The presence of the methyl groups also affects the conformation of molecules which, in solution and in crystalline form, have the stretched geometry. This means that our hemiaminals in solution have the RS/SR configuration. The current study revealed the enormous influence of the environment on the reaction course and its efficiency. In this respect, the solvent polarity, the presence of water and its catalytic performance are important factors. A simple relationship between temperature and the product yield as well as the metathesis phenomena observed in this work led to the conclusion that the first stage of condensation—the creation of a hemiaminal—is an exothermic process, while the second—a Schiff base formation—is an endothermic process.
